# The Association between Mortality-to-Incidence Ratios and Health Expenditures in Brain and Nervous System Cancers

**DOI:** 10.3390/ijerph16152739

**Published:** 2019-07-31

**Authors:** Tsung-Han Lee, Wen-Wei Sung, Lung Chan, Hsiang-Lin Lee, Sung-Lang Chen, Yu-Hui Huang, Aij-Lie Kwan

**Affiliations:** 1 Division of Neurosurgery, Department of Surgery, Kaohsiung Chang Gung Memorial Hospital and Chang Gung University College of Medicine, Kaohsiung 83301, Taiwan; 2 Graduate Institute of Medicine, College of Medicine, Kaohsiung Medical University, Kaohsiung 80708, Taiwan; 3 Institute of Medicine, Chung Shan Medical University, Taichung 40201, Taiwan; 4 School of Medicine, Chung Shan Medical University, Taichung 40201, Taiwan; 5 Department of Urology, Chung Shan Medical University Hospital, Taichung 40201, Taiwan; 6 Department of Surgery, Chung Shan Medical University Hospital, Taichung 40201, Taiwan; 7 Department of Physical Medicine & Rehabilitation, Chung Shan Medical University Hospital, Taichung 40201, Taiwan; 8 Department of Neurosurgery, Kaohsiung Medical University Hospital, Kaohsiung 80756, Taiwan

**Keywords:** brain cancer, nervous system cancer, mortality, incidence, mortality-to-incidence ratio, expenditure, human development index

## Abstract

Mortality-to-incidence ratios (MIRs) are alternative parameters used to evaluate the prognosis of a disease. In addition, MIRs are associated with the ranking of health care systems and expenditures for certain types of cancer. However, a lack of association between MIRs and pancreatic cancer has been noted. Given the poor prognosis of brain and nervous system cancers, similar to pancreatic cancer, the relation of MIRs and health care disparities is worth investigating. We used the Spearman’s rank correlation coefficient (CC) to analyze the correlation between the MIRs in brain and nervous system cancers and inter-country disparities, including expenditures on health and human development index. Interestingly, the MIRs in brain and nervous system cancers are associated with the human development index score (N = 157, CC = −0.394, *p* < 0.001), current health expenditure (CHE) per capita (N = 157, CC = −0.438, *p* < 0.001), and CHE as percentage of gross domestic product (N = 157, CC = −0.245, *p* = 0.002). In conclusion, the MIRs in the brain and nervous system cancer are significantly associated with health expenditures and human development index. However, their role as an indicator of health disparity warrants further investigation.

## 1. Introduction

The incidence of brain and nervous system cancers varies, which is a function of occupation and age distribution; moreover, these cancers have a high mortality rate and are rapidly becoming serious health issues worldwide. A total of 296,851 new cases (age-standardized rate, ASR = 3.9 and 3.1, male and female, respectively) of brain and other nervous system cancers have been diagnosed, and 241,037 cancer deaths (ASR = 3.2 and 2.3, male and female, respectively) worldwide were recorded in 2018 [1]. In the United States, brain and nervous system cancers are the leading causes of cancer-related death in men under the age of 40 and in women under the age of 20 [2]. Brain and nervous system cancers can occur at any age, but some studies have shown that they are most common in children (aged 0 to 14 years) and adolescents (aged 15 to 19 years) [2]. Studies have also shown that brain cancer is associated with patients’ occupation; for instance, steel foundry workers, farmers and pesticide users are at an increased risk of brain cancer [3,4,5]. In addition, evidence supports that brain tumors are associated with ionizing radiation, which is generally emitted from cancer treatments and from nuclear weapons. Exposure to moderate radiation doses (i.e., <1 sievert) is significantly associated with increased incidence of neurological tumors [6].

In recent years, many breakthroughs were achieved in brain and nervous system cancer treatment. Surgical resection has proven to be an effective traditional treatment, and chemotherapy or radiation therapy can kill any remaining cancer cells after surgery to reduce the risk of cancer recurrence [7,8]. Targeted therapy, immunotherapy and nanomedicine therapy are novel, curative treatment methods used to treat brain and nervous system cancers [9,10,11]. Considerable progress has been achieved in these therapies over the last couple of years, and such progress has demonstrated a considerable potential for the development of more effective and less toxic therapeutic interventions. Although the above treatments for brain and nervous system cancers have improved considerably, the number of deaths has remained high, as shown in recent global analyses [1,2], indicating that precancerous prevention may be an effective method to control the morbidity of brain and nervous system cancers.

An innovative parameter used to assess the prognosis of a specific disease is the mortality-to-incidence ratio (MIR), which has been a prime factor for the long-term success of cancer surveillance and for the efficacy of cancer control programs [12,13,14,15]. Because the incidence of brain and nervous system cancers varies as a function of occupation and age distribution, we conjecture that the human development index score and health expenditure affect the incidence, mortality rate or MIRs of these cancers. Because this topic has not yet been investigated, we conducted this study to gain a better understanding of the incidence, mortality and MIR of brain and nervous system cancers.

## 2. Materials and Methods

Data on cancer incidence and mortality were obtained from the GLOBOCAN 2018 database, which is maintained by the International Agency for Research on Cancer (https://www.iarc.fr/). The International Statistical Classification of Diseases and Related Health Problems 10th Revision (ICD-10) of C70–72 (brain, central nervous system) was collected according to the method of GLOBOCAN. A total of 185 countries are included in the database. Human development index was obtained from the Human Development Reports database of the United Nations Development Programme (http://hdr.undp.org/). Parameters including current health expenditure (CHE) per capita, and CHE as percentage of gross domestic product (CHE/GDP) were obtained from the WHO statistics report (https://www.who.int/; World Health Statistics 2018). The data acquisition methods used in this study were described previously [16,17]. MIR is defined as the ratio of crude mortality rate to incidence [16,18,19,20]. The selected countries were further screened based on missing data (N = 12, no data available in World Health Statistics 2018; N = 1, no data on human development index), on having a crude rate of zero for incidence or mortality (N = 8), or on being an outlier according to box-and-whisker diagram of the MIR (N = 7). Ultimately, 157 countries were included in the analyses. 

The statistical analysis methods used in this study were described previously [16,19,20,21]. We evaluated the association between the MIRs and variables with Spearman’s rank correlation coefficient (CC) by using SPSS statistical software version 15.0 (SPSS, Inc., Chicago, IL, USA). *p* values of <0.05 indicated statistical significance. Scatter plots were generated using Microsoft Excel 2016.

### 2.1. Research Involving Human Participants

All data were obtained from the global statistics of GLOBOCAN (https://www.iarc.fr/), United Nations Development Programme, Human Development Reports (http://hdr.undp.org/), and WHO statistics report (https://www.who.int/). This work is an analytic epidemiological study, and we did not perform any intervention on human participants.

### 2.2. Informed Consent

All data were obtained from the global statistics of GLOBOCAN (https://www.iarc.fr/), United Nations Development Programme, Human Development Reports (http://hdr.undp.org/), and WHO statistics report (https://www.who.int/). This research is an analytic epidemiological study that involved no intervention on human participants, so no informed consent was required. 

### 2.3. Declarations

Ethics committee approval and consent to participate are not applicable. All data were obtained from the global statistics of GLOBOCAN (https://www.iarc.fr/), United Nations Development Programme, Human Development Reports (http://hdr.undp.org/), and WHO statistics report (https://www.who.int/). This work is an analytic epidemiological study, and we did not perform any intervention on human participants. We confirm that this study complies with the national guidelines (http://law.moj.gov.tw/LawClass/LawAll.aspx?PCode=L0020162).

## 3. Results

### 3.1. Incidence and Mortality of Brain and Nervous System Cancers According to Region 

The incidence and mortality rates, rank, percentage of brain and nervous system cancers in all types of cancer and cumulative risk are summarized in Table 1. The total number of cases and deaths worldwide were 296,851 and 241,037, respectively, during the survey period. Among all types of cancer, brain and nervous system cancers accounted for 1.84% of the total number of cases and 2.73% of the total number of deaths. In terms of regions, Africa had the lowest incidence and mortality cumulative risk (0.18 and 0.17, respectively), whereas the highest values (0.56 and 0.44, respectively) were observed in Europe. 

### 3.2. Incidence and Mortality of Brain and Nervous System Cancers According to Country

Table 2 summarizes the human development index, CHE and MIR of brain and nervous system cancers in 40 representative countries selected according to their ranking for incidence crude rate. The six countries with an incidence crude rate higher than 10 were Serbia (10.3), Croatia (10.3), Greece (10.4), Bosnia and Herzegovina (10.5), Lithuania (11.0) and North Macedonia (12.0). Three of these countries were also among those with a crude mortality rate higher than 8.0, with Lithuania (7.4), Greece (7.7) and Croatia (7.9) being the three exceptions. The other country with a mortality rate higher than 8.0 was Bulgaria (8.8). The calculated MIRs are also presented in Table 2. The four countries with MIRs higher than or equal to 0.90 were Georgia (0.90), Armenia (0.93), Denmark (0.97) and Bulgaria (1.00). Only Sweden has an MIR that is lower than 0.65 (i.e., 0.53). 

### 3.3. Association among Human Development Index, CHE Per Capita, CHE/GDP, and MIR in Different Countries

**Figure 1:**
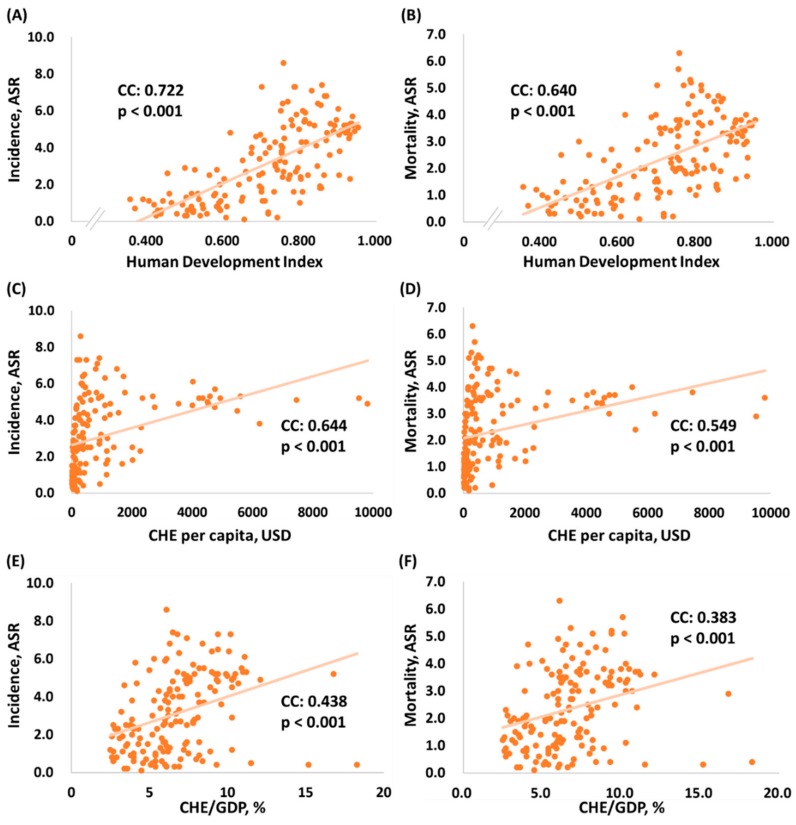
Association among human development index, current health expenditure and age-standardized rate of (**A**,**C**,**E**) incidence and (**B**,**D**,**F**) mortality in brain and nervous system cancers (N = 157). CHE: current health expenditure; CHE/GDP: current health expenditure as percentage of gross domestic product; ASR: age-standardized rate.

The linear correlation among human development index, CHE per capita, CHE/GDP, age-standardized incidence, and mortality rates in 157 different countries are shown in Figure 1. Figure 1A and 1B present the human development index, which is significantly associated with the ASR of incidence/mortality (N = 157, CC = 0.722, *p* < 0.001, Figure 1A; N = 157, CC = 0.640, *p* < 0.001, Figure 1B, respectively). Figure 1C,D show that CHE per capita is markedly associated with ASR of incidence/mortality (N = 157, CC = 0.644, *p* < 0.001, Figure 1C; N = 157, CC = 0.549, *p* < 0.001, Figure 1D, respectively). Moreover, CHE/GDP is significantly associated with ASR of incidence/mortality as shown in Figure 1E,F (N = 157, CC = 0.438, *p* < 0.001, Figure 1E; N = 157, CC = 0.383, *p* < 0.001, Figure 1F, respectively). 

**Figure 2:**
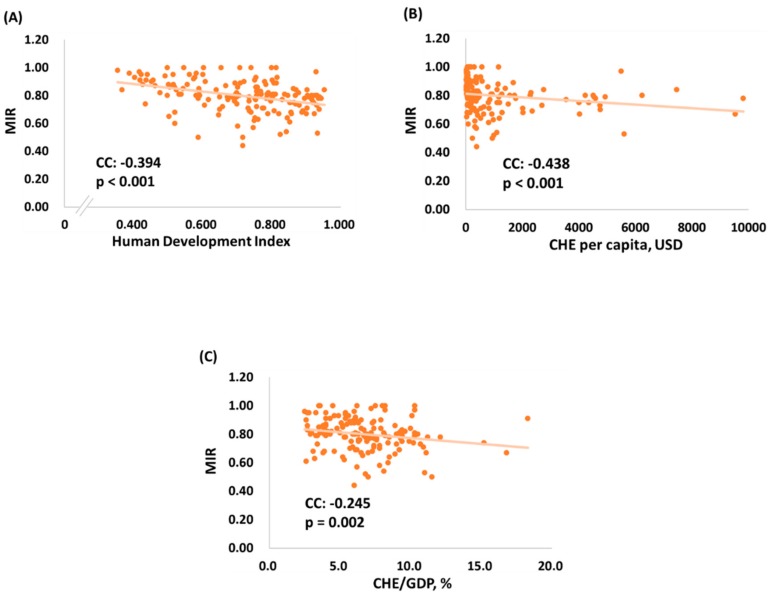
The association of (**A**) human development index, (**B**) current health expenditure per capita (CHE per capita), (**C**) current health expenditure as percentage of gross domestic product (CHE/GDP) and mortality-to-incidence ratio for brain and nervous system cancers (N = 157).

In Figure 2A–C, human development index, CHE per capita and CHE/GDP are all significantly associated with the MIRs of the 157 countries. The negative correlation between human development index and MIRs (N = 157, CC = −0.394, *p* < 0.001, Figure 2A) indicates that the countries with a higher level of development have a lower MIR. In addition, CHE per capita and CHE/GDP showed a negative correlation (N = 157, CC = −0.438, *p* < 0.001, Figure 2B; N = 157, CC = −0.245, *p* < 0.002, Figure 2C, respectively), indicating that the countries with a higher CHE per capita and CHE/GDP have a lower MIRs for brain and nervous system cancers. 

## 4. Discussion

The results show that the crude rates and ASRs of incidence and mortality are higher in more developed regions than in less developed regions. We speculate that this trend is attributed to higher exposure of individuals in more developed regions to risk factors (e.g., exposure to more carcinogens) that are associated with brain and nervous system cancers. Ionizing radiation is one such carcinogen, which is used in various applications that are common in more developed countries; these applications include nuclear weapons, nuclear power plants, co-contaminated buildings, and medical examinations and procedures. Exposure to ionizing radiation can increase the risk of brain and nervous system cancers [6,16,17]. In addition, although the association between electromagnetic fields and brain cancer remains uncertain, some studies have indicated that long-term use of mobile phones might increase the risk of intracranial tumors [18].

Human development index, CHE per capita and CHE/GDP are all markedly positively associated with the ASR of incidence/mortality. This finding indicates that incidence and mortality rates are higher in countries with a better economy. These associations are expected, and as discussed earlier, the incidence and mortality rates of brain and nervous system cancers are higher in more developed regions, which are usually more willing to invest more in health, leading to a higher human development index ranking, CHE per capita and CHE/GDP [19].

A negative correlation was observed between human development index ranking and MIR for brain and nervous system cancers in 157 different countries, that is, the countries with a lower human development index ranking have a higher MIR. This correlation may indicate that countries with higher human development index rankings tend to have better public health policies and tools to screen and treat brain and nervous system cancers. Image testing is one of the critical parameters used to evaluate tumors and to plan treatments. Magnetic resonance imaging is commonly used to diagnose brain tumors; other imaging instruments, such as computed tomography and positron emission tomography, are also used to screen brain and nervous system cancers [20,21]. These screening instruments are more readily available in countries with higher health care rankings. Overall, early detection combined with effective treatment decreases MIR, which makes sense based on the results.

To our best knowledge, our study is the first to investigate the association among MIR of brain and nervous system cancer, human development index, CHE per capita and CHE/GDP. However, our study has some limitations. First, we did not include the countries with poor data quality or those without available data quality assessments, which might cause misleading findings on MIR. Second, because we collected cross-sectional data for one year only, these data may not accurately describe the actual trend of the disease or they may not allow us to infer causality. Third, no detailed categories of cancer types were analyzed in this study. Despite these limitations, our research shows that in more developed regions and countries with higher CHE/GDP, the incidence and mortality rates for brain and nervous system cancers are higher. 

## 5. Conclusions

MIRs for brain and nervous system cancers are significantly associated with health expenditures and human development index. Considering the limitation of using MIR to predict cancer prognosis, their role and causal relationship are still debatable.

## Figures and Tables

**Table 1:** Summary of the number of cases, rank and percentage of brain and nervous system in all cancers in the selected regions.

Region	New Cases				Deaths			
	Number	Rank	% of All Cancers ^1^	Cum. Risk ^2^	Number	Rank	% of All Cancers ^1^	Cum. Risk ^2^
All regions	296,851	18	1.84	0.31	241,037	13	2.73	0.30
Africa	16,956	16	1.84	0.18	14,225	15	2.35	0.17
Asia	156,217	16	1.91	0.31	129,483	12	2.52	0.27
Europe	64,639	17	1.72	0.56	53,027	12	2.94	0.44
Latin America and the Caribbean	29,539	14	2.36	0.42	22,312	10	3.70	0.33
North America	27,062	19	1.50	0.52	19,973	10	3.16	0.36
Oceania	2438	19	1.42	0.47	2,017	12	3.21	0.40

^1^ % of all cancers: percentage of brain and nervous system cancers in all types of cancer; ^2^ cum. risk: cumulative risk.

**Table 2:** Representative data for human development index, current health expenditure, cancer incidence, cancer mortality and mortality-to-incidence ratio in brain and nervous system cancers in selected countries according to ranking of incidence (N = 40).

Country	Human Development Index		Current Health Expenditure		Incidence			Mortality			Mortality-to-Incidence Ratio
	Score	Rank	Per Capita	/GDP (%) ^1^	ASR ^2^	CR ^3^	Cum. Risk ^4^	ASR ^2^	CR ^3^	Cum. Risk ^4^	
North Macedonia	0.757	80	295	6.1	8.6	12.0	1.0	6.3	9.6	0.8	0.80
Lithuania	0.858	35	923	6.5	7.4	11.0	0.8	4.7	7.4	0.6	0.67
Bosnia and Herzegovina	0.768	77	431	9.4	6.5	10.5	0.8	5.1	8.7	0.7	0.83
Greece	0.870	31	1505	8.4	6.8	10.4	0.8	4.6	7.7	0.6	0.74
Croatia	0.831	46	852	7.4	7.1	10.3	0.8	4.7	7.9	0.6	0.77
Serbia	0.787	67	491	9.4	7.3	10.3	0.8	5.2	8.3	0.6	0.81
Poland	0.865	33	797	6.3	6.8	9.7	0.7	4.5	7.3	0.6	0.75
Albania	0.785	68	266	6.8	7.3	9.7	0.8	5.3	7.6	0.7	0.78
Portugal	0.847	41	1722	9.0	6.4	9.5	0.7	4.5	7.6	0.5	0.80
Republic of Moldova	0.700	112	186	10.2	7.3	9.2	0.7	5.1	6.8	0.6	0.74
Bulgaria	0.813	51	572	8.2	5.5	8.8	0.6	5.1	8.8	0.6	1.00
France	0.901	24	4026	11.1	6.1	8.5	0.7	3.7	5.7	0.4	0.67
Slovakia	0.855	38	1108	6.9	6.3	8.5	0.7	4.2	6.4	0.5	0.75
Montenegro	0.814	50	382	6.0	5.9	8.5	0.7	4.9	7.6	0.7	0.89
Romania	0.811	52	442	5.0	5.4	8.3	0.6	4.1	6.7	0.5	0.81
Italy	0.880	28	2700	9.0	5.3	8.2	0.6	3.3	6.0	0.4	0.73
Slovenia	0.896	25	1772	8.5	5.5	8.2	0.6	3.5	6.4	0.5	0.78
Armenia	0.755	83	366	10.1	6.4	8.1	0.7	5.7	7.5	0.7	0.93
Germany	0.936	5	4592	11.2	5.3	7.8	0.6	3.6	6.1	0.4	0.78
Spain	0.891	26	2354	9.2	5.2	7.7	0.6	3.2	5.3	0.4	0.69
Belarus	0.808	53	352	6.1	6.0	7.7	0.6	3.7	5.3	0.4	0.69
Cuba	0.777	73	826	10.9	5.5	7.7	0.6	3.7	5.5	0.4	0.71
Hungary	0.838	45	894	7.2	5.2	7.4	0.6	3.7	6.0	0.4	0.81
Czechia	0.888	27	1284	7.3	4.9	7.3	0.5	3.0	5.0	0.4	0.68
Ireland	0.938	4	4757	7.8	5.7	7.3	0.6	3.7	5.1	0.5	0.70
Switzerland	0.944	2	9818	12.1	4.9	7.3	0.6	3.6	5.7	0.4	0.78
United Kingdom	0.922	14	4356	9.9	5.2	7.2	0.6	3.4	5.4	0.4	0.75
Belgium	0.916	17	4228	10.5	5.2	7.1	0.5	3.8	5.7	0.4	0.80
Sweden	0.933	7	5600	11.0	5.3	7.0	0.5	2.4	3.7	0.3	0.53
Estonia	0.871	30	1112	6.5	4.8	6.9	0.5	3.9	5.8	0.5	0.84
Georgia	0.780	70	281	7.9	5.2	6.9	0.6	4.4	6.2	0.5	0.90
Finland	0.920	15	4005	9.4	4.8	6.8	0.5	3.2	5.1	0.4	0.75
Australia	0.939	3	4934	9.4	5.2	6.8	0.6	3.7	5.4	0.5	0.79
Austria	0.908	20	4536	10.3	4.9	6.8	0.5	3.3	5.4	0.4	0.79
Norway	0.953	1	7464	10.0	5.1	6.8	0.5	3.8	5.7	0.5	0.84
Denmark	0.929	11	5497	10.3	4.5	6.8	0.5	4.0	6.6	0.5	0.97
Netherlands	0.931	10	4746	10.7	4.7	6.7	0.5	3.0	4.9	0.4	0.73
Canada	0.926	12	4508	10.4	5.0	6.6	0.5	3.4	5.3	0.4	0.80
New Zealand	0.917	16	3554	9.3	4.9	6.5	0.5	3.5	5.0	0.4	0.77
United States of America	0.924	13	9536	16.8	5.2	6.4	0.5	2.9	4.3	0.4	0.67

^1^ Current health expenditure as percentage of gross domestic product; ^2^ ASR: age-standardized rate; ^3^ CR: crude rate; ^4^ cum. risk: cumulative risk.

## Abbreviations

ASR:	age-standardized rate
CC:	correlation coefficient
CHE:	current health expenditure
CHE/GDP:	current health expenditure as percentage of gross domestic product
CR:	crude rate
cum. risk:	cumulative risk
MIR:	mortality-to-incidence ratio
WHO:	World Health Organization
